# The challenge of HLA donor specific antibodies in the management of pancreatic islet transplantation: an illustrative case-series

**DOI:** 10.1038/s41598-022-16782-3

**Published:** 2022-07-21

**Authors:** Mehdi Maanaoui, Mikael Chetboun, Isabelle Top, Vincent Elsermans, Julie Kerr-Conte, Kristell Le Mapihan, Frederique Defrance, Valéry Gmyr, Thomas Hubert, Myriam Labalette, Marc Hazzan, Marie-Christine Vantyghem, François Pattou

**Affiliations:** 1 Department of Nephrology, Service de Néphrologie, CHU de Lille, Hôpital Huriez, 59037 Lille, France; 2 Inserm, CHU Lille, Institut Pasteur Lille, University in Lille, U1190 - EGID, 59000 Lille, France; 3 Department of General and Endocrine Surgery, CHU Lille, 59000 Lille, France; 4 Service d’Immunologie, CHU Lille, 59000 Lille, France; 5 Plateforme de Biothérapie, CHU Lille, 59000 Lille, France; 6 Department of Endocrinology, Diabetology, and Metabolism, CHU Lille, 59000 Lille, France

**Keywords:** Type 1 diabetes, Allotransplantation

## Abstract

Islet transplantation is a unique paradigm in organ transplantation, since multiple donors are required to achieve complete insulin-independence. Preformed or de novo Donor Specific Antibodies (DSA) may target one or several donor islets, which adds complexity to the analysis of their impact. Adult patients with type 1 diabetes transplanted with pancreatic islets between 2005 and 2018 were included in a single-center observational study. Thirty-two recipients with available sera tested by solid-phase assays for anti-HLA antibodies during their whole follow-up were analyzed. Twenty-five recipients were islet-transplantation-alone recipients, and 7 islet-after-kidney recipients. Seven recipients presented with DSA at any time during follow-up (two with preformed DSA only, one with preformed and de novo DSA, 4 with de novo DSA only). Only islet-transplantation-alone recipients presented with de novo DSA. Three clinical trajectories were identified according to: 1/the presence of preformed DSA, 2/early de novo DSA or 3/late de novo DSA. Only late de novo DSA were associated with unfavorable outcomes, depicted by a decrease of the β-score. Islet transplantation with preformed DSA, even with high MFI values, is associated with favorable outcomes in our experience. On the contrary, de novo DSA, and especially late de novo DSA, may be associated with allograft loss.

## Introduction

Islet transplantation is a cell therapy which has been shown to be an effective treatment to reverse type 1 diabetes^1^. Using a pancreas procured from a deceased donor, islet cells are isolated and infused, most of the time in the recipient’s portal vein. The transplantation of one donor islet preparation allows a reduction of 30 to 50% of insulin needs, with approximately 20% of recipients reaching insulin-independence^2–4^. Consequently, multiple injections are required, up to three, to achieve long-term complete insulin-independence^5^. Indeed, determinants of islet transplantation success rely on the primary-graft function^6^. The recipient immune system is then exposed to multiple allogenic stimuli, which increases the risk of HLA antibody formation against islet cells, i.e. Donor Specific Antibodies (DSA). In the general context of solid organ transplantation, such as the kidney or the heart, antibody-mediated rejection is one of the leading cause of graft failure^7^. DSA antibodies can either be present at the time of transplantation, termed “preformed” DSA, or can appear after transplantation, termed “de novo” DSA. In recent years, detection of HLA antibodies has become increasingly more sensitive with the introduction of multiple bead–based technologies. Luminex technology helps to define precisely against which HLA antigens the antibodies are targeted, and gives a semi-quantitative value estimator of the abundance of antibodies, measured by the Mean Fluorescence Intensity (MFI)^8^. The impact of rejection and DSA in islet transplantation remains however debated, as, unlike other organs, no validated method exists to monitor rejection. Mechanisms of rejection, however, are supposed to be mediated more by cellular auto- or allo-reactivity, than by humoral auto- or allo-immunity^9,10^. Thus, in most allocation programs, no specific recommendations are given regarding the presence of HLA antibodies at the time of islet transplantation^1,11^ and no HLA matching is also currently required to perform islet transplantation. Moreover, considering the multiplicity of sequential infusion, de novo DSA can appear after transplant and target one or several of the islet donors. Very few studies exist on the topic and data are controversial, as the largest studies performed found either a deleterious impact only for de novo DSA^12^, or no impact at all^13^. To improve the management of DSA antibodies after islet transplantation, we propose to describe the different clinical outcomes according to the DSA status after islet transplantation in a retrospective case-series.

## Methods

### Patients and study design

Every adult with type 1 diabetes, with a negative C-peptide, having received an islet transplantation in a single university hospital, between January 2005 and December 2016 was included in this observational study. Indications for islet transplantation were either the presence of severe hypoglycemic episodes or an end-stage renal disease requiring kidney transplantation. Only patients with available sera before and during their whole follow-up tested by Luminex were analyzed. The end of follow-up was defined as December 2019 or at the time of islet graft loss. The study data were obtained from the patient’s clinical records (NCT00446264, NCT01123187, NCT01148680).

### Ethical statement

This study was performed according to the Declaration of Helsinki and the Declaration of Istanbul. No organs were procured from prisoners. As the French Biomedical Agency regulates the allocation system in France, every organ was allocated by the Agency and transplanted in Lille, France (Centre Hospitalier Universitaire, Lille). Ethical committee was bypassed, according to French laws and the local institutional review board (Centre Hospitalier Universitaire, Lille)^14^, as the study was monocentric and observational. Informed consent was obtained from all subjects. No subjects under 18 were involved in the study. Once fully pseudonymized, the dataset was processed under French and EU data protection laws and regulations (Commission Nationale de l’Informatique et des Libertés, CNIL).

### Islet isolation and allogenic transplantation procedure

Pancreata from deceased donors were processed within 12 h of procurement, and islets were isolated with a slightly modified standard automated method as described previously^15^. Up to three sequential ABO-compatible islet infusions were performed after a negative lymphocytotoxicity-based crossmatch over a 3-month period. Access to the portal vein was gained under general anesthesia by percutaneous catheterization of a peripheral portal branch under ultrasound guidance or by a surgical mini-laparotomy with catheterization of a proximal mesenteric vein. Islet recipients were treated according to two separate immunosuppressive regimens. Protocol A consisted in an induction therapy including five-doses of daclizumab (1 mg/kg). Maintenance consisted of oral therapy with tacrolimus, target trough levels at 5–8 ng/mL the first year, then 3-5 ng/mL, and sirolimus, target trough levels at 12–15 ng/mL the first three months, 7–10 ng/mL the first year, then 5–7 ng/mL. Protocol B consisted in an induction therapy including etanercept (50 mg the day of islet infusion then 25 mg at days 3, 7 and 10 after islet infusion), and thymoglobulin during the first infusion, administered 2 days before (0.5 mg/kg), one day before (1 mg/kg), the day (1.5 mg/kg) and two days after (1.5 mg/kg) islet infusion. One hour before the first thymoglobulin infusion, 2 mg/kg methylprednisolone was administrated intravenously and pentoxifillin (400 mg twice per day for 5 days) was started. For the second and third islet infusions, basiliximab (20 mg intravenously) was administered 2 h before and 4 days after transplantation. Maintenance consisted of oral tacrolimus (1 mg twice per day), target trough levels at 9–13 ng/mL for 3 months after transplantation that were decreased to a target of 6–10 ng/mL and Mycophenolate mofetil (1 g twice per day).

### HLA antibody monitoring

Patients were selected according to a complete work-up of HLA antibodies during their follow-up. Standard follow-up consisted in HLA antibody screening every 3 months while on the waiting list, at the day of transplantation, then at day 15, month 1 and every month until the last islet infusion. HLA screening was performed 3 months after the final islet infusion, and finally once a year until the end of follow-up, or in case of an event. Class I and II anti-HLA antibodies were defined by the presence or absence of class I and II anti-HLA antibodies by the LABScreen Mixed Luminex flow bead assay (One Lambda). In case of positivity, specificities and MFI were determined according to the LABScreen Single Antigen Luminex flow bead assay (One Lambda). DSA were defined as positive and clinically-relevant if a minimum MFI value of 1000 was reached. High-MFI values were considered when over 3000. Early de novo DSA was considered if the time between injection and DSA occurrence was below 12 months post-injection.

### Autoantibody testing

Patients were monitored at baseline and during follow-up (every month the first year, then every year) for the main autoantibodies involved in type 1 diabetes, i.e. anti-Glutamic Acid Decarboxylase (GAD), anti-Insulin AutoAntibodies (IAA) and anti-Islet Cell Autoantibodies (ICA).

### Definition and outcomes

The metabolic β-score was calculated during the follow-up, as a composite index ranging from 0 (no graft function) to 8 (excellent graft function) 16, and included the following variables: fasting plasma glucose, stimulated C-peptide, daily insulin and HbA1c. Primary graft function was evaluated within one month of the last islet infusion, and considered as optimal if the β-score was ≥ 7, as previously published by our group 6. Graft loss was considered when C-peptide upon stimulation was undetectable (< 0.3 ng/ml).

### Statistical analysis

The median and first-third quartile were used for continuous data, whereas categorical variables were summarized as counts and proportions. Graphs and Figures were designed with GraphPad Prism 9.0 (GraphPad Software ®, Inc., San Diego, CA). We analyzed the data by fitting a mixed model as implemented in GraphPad Prism 8.0. This mixed model uses a compound symmetry covariance matrix, and is fit using Restricted Maximum Likelihood (REML).

## Results

### Study patients

**Figure 1:**
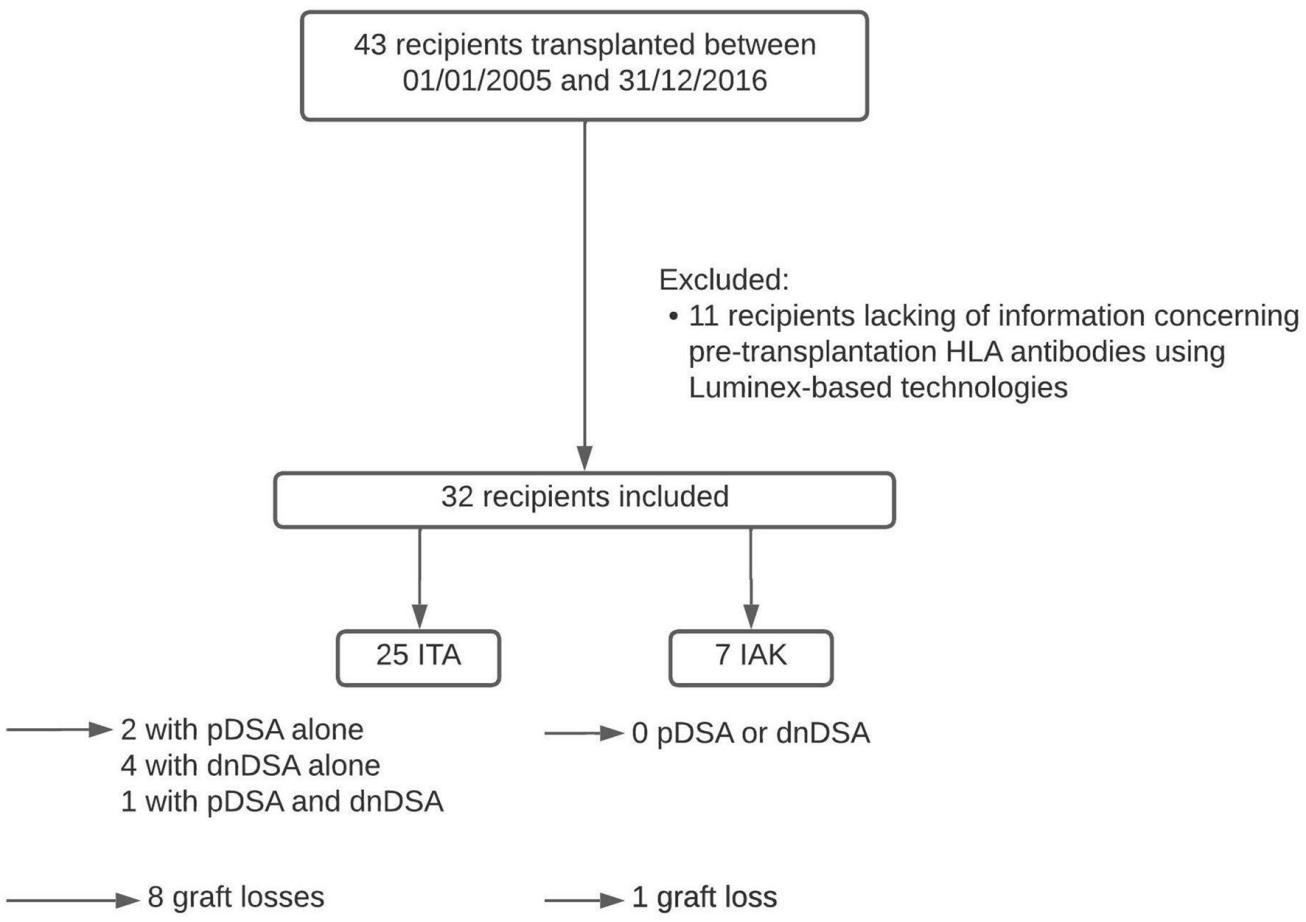
Flow-chart of the study. *ITA* islet-transplantation alone; *IAK* islet-after-kidney; *pDSA* preformed Donor Specific Antibodies; *dnDSA* de novo Donor Specific Antibodies.

**Figure 2:**
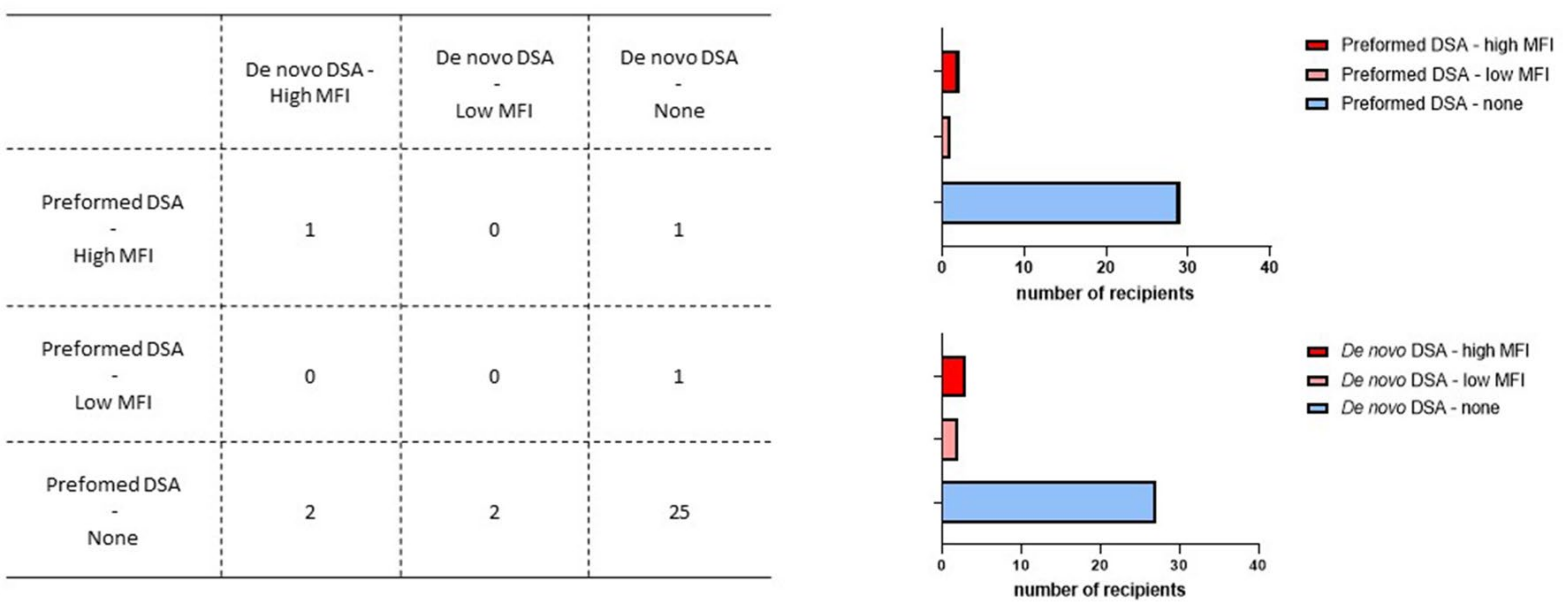
Distribution of donor specific antibodies in a cohort of type 1 diabetic pancreatic islets recipients. There are nine possible combinations considering the presence or not of preformed or de novo DSA and the level of MFI (left Table). High MFI was considered with a cut-off of 3000. A majority of recipients does not present with either preformed or de novo DSA (preformed DSA: top right graph; de novo DSA below: right graph). DSA: Donor Specific Antibodies. *MFI* Mean Fluorescence Intensity.

Thirty-two pancreatic islet recipients matched the criteria of inclusion with complete pre- and post-transplantation Luminex follow-up (See Fig. 1). Baseline characteristics are described Table 1. Median time of follow-up was 66.0 months (48.0–108.0). The median number of islet injections was 3 (3–3). Twenty-five recipients (78.1%) were islet-transplantation-alone recipients (ITA) and 7 recipients (21.9%) were islet-after kidney recipients (IAK). Twenty-one recipients (65.6%) had an optimal graft function one month after their last islet infusion. The median number of total A-B-DR-DQ HLA mismatches per recipient was 17.0 (15.8–19.0). Nineteen (59.4%) out of the 32 recipients benefited from the Edmonton protocol immunosuppressive regimen (Protocol A). Among these 32 recipients, 7 patients presented with DSA: 2 with preformed DSA alone, 4 with de novo DSA alone, and 1 with preformed and de novo DSA (Fig. 2). Considering preformed DSA, the number of islet injections targeted went from 2 to 3, and the number of HLA antigens targeted from 3 to 15 (Table 2). Considering de novo DSA, the median time of emergence was 17.0 months (7.0–90.0) The number of islet injections targeted by de novo DSA went from 1 to 2, and the number of HLA antigens targeted from 2 to 3. HLA typing of donor and recipients are depicted in Supplemental Table 1. We evaluated then the difference between negative and positive DSA recipients regarding metabolic outcomes. Overall, we did not find any significant effect of the presence of DSA on the evolution of the Beta-Score at 1 year, 3 years and 5 years (fixed effect between DSA negative and positive recipient: −1.07, 95% CI: from −3.05 to 0.92, *p* = 0.16), or the evolution of HBA1c (fixed effect between DSA negative and positive recipient: 0.55, 95% CI: from −0.26 to 1.36, *p* = 0.18) (Supplemental Fig. 1). We also compared tacrolimus and sirolimus trough levels between DSA positive and negative recipients, and did not find any significant difference (Supplemental Fig. 2A).

**Table 1:** Baseline characteristics of the cohort.

	Overall recipients (n = 32)
Median time of follow-up (months): median (IQR)	66.0 (48.0–108.0)
Recipient age (years): median (IQR)	47.50 (51.5–56.2)
Recipient sex (Male): n (%)	15 (46.8%)
Recipient BMI (kg/m^2^): median (IQR)	22.8 (20.4–25.1)
Exogenous insulin requirements (IU/kg per day): median (IQR)	0.54 (0.41–0.63)
No. of severe hypoglycemic events in previous year: median (IQR)	2 (0–3)
Diabetes duration (years)	30.0 (23.5–37.25)
Islet-after-Kidney recipient: n (%)	5 (15.6%)
Number of islet infusions: median (IQR)	3 (3–3)
Total tissue volume (mL): median (IQR)	11.9 (9.3–14.4)
Total islet mass (10^3^ IEQ/kg): median (IQR)	13.3 (11.4–14.5)
Islet viability (%): median (IQR)	94.2 (91.9–95.9)
Islet purity (%): median (IQR)	50.5 (46.3–56.1)
Islet function (GSIS): median (IQR)	1.9 (1.6–2.4)
Optimal primary graft function: n (%)	21 (65.6%)
Mean number of HLA mismatches (ABDRDQ): median (IQR)	17.0 (15.8–19.0)
**Induction immunosuppressive regimen: n (%)**	
Thymoglobulin + Etanercept (TRIMECO)	13 (41.6)
Anti-IL2R (EDMONTON)	19 (59.4)
**Maintenance immunosuppressive regimen**	
Tac + MMF	13 (41.6)
Tac + SRL	19 (59.4)

*BMI* Body-Mass index, *GSIS* Static Glucose-stimulated Insulin Secretion, *HLA* Human Leukocyte Antigen, IL2R: Interleukin 2–Receptor; *IU* insulin unit, *MMF* Mycophenolate Mofetil, *SRL* Sirolimus. *Tac* Tacrolimus.

**Table 2:** Characteristics of HLA Donor-Specific Antibodies in the cohort.

	Immunosuppressive regimen	Preformed DSA	Number of islet infusions targeted	Total number of antigens targeted	Maximum MFI	de novo DSA	Time of appearance after the 1st injection (mo.)	Number of islet infusions targeted	Total number of antigens targeted	Maximum MFI
Patient#1	Edmonton	yes	3	15	21,000	no				
Patient#2	Edmonton	yes	2	3	2500	no				
Patient#3	Trimeco	yes	3	5	9500	yes	7	1	1	4300
Patient#5	Edmonton	no				yes	6	1	2	2900
Patient#4	Trimeco	no				yes	17	1	3	4800
Patient#6	Edmonton	no				yes	90	2	2	1000
Patient#7	Trimeco	no				yes	105	2	3	20,000

*DSA*  Donor Specific-Antibodies, *MFI*  Mean Fluorescence IntensityAdditional supporting information may be found online in the Supporting Information section.

Considering the low number of events and in order to provide a deeper analysis of these recipients presenting with DSA, we evaluate then clinical trajectories according to the type and time of DSA occurrence.

### HLA antibodies in islet-transplantation alone recipients

Impact of preformed Donor Specific Antibodies with high or low fluorescence intensities.

**Figure 3:**
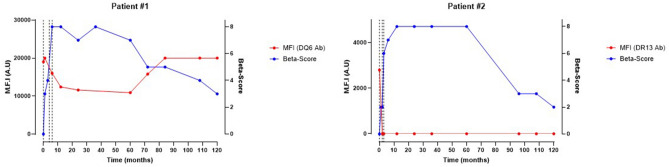
Evolution of β-Score over time in type 1 diabetic pancreatic islet recipients presenting with high or low preformed Donor Specific Antibodies. Patient#1 (left panel) presented with high-MFI preformed DSA targeting every islet transplantation. Patient#2 (right panel) presented with a low-MFI preformed DSA targeting two islet donors (only one presented). Vertical dash lines represent every pancreatic islet injection. *Ab* Antibody; *MFI* Mean-Fluorescence intensity.

Patient#1 was a 55-year-old woman, with a 47 year-history of type 1 diabetes (Fig. 3, left panel). She benefited from a first infusion of 7194 islet-equivalent/kg (IEQ/kg) with Protocol A immunosuppressive regimen. At the time of the first infusion, the patient had preformed DSA against 4 HLA antigens: A2, B62, B44 and DR4, with respective MFI of 10,850, 1330, 1700 and 8350. The second islet infusion was performed 4 months later, with 3115 IEQ/kg. No de novo DSA appeared between the 1st and the 2nd islet infusions. At the time of the 2nd infusion, the patient had preformed DSA against 5 HLA antigens: A1, B8, B62, DR4, with respective MFI of 10,000, 13,000, 2000 and 7500. Finally, the 3rd islet infusion was performed two months later, with 5540 IEQ/kg. No de novo DSA appeared between the 2nd and the 3rd islet infusions. At the time of the 3rd infusion, the patient had preformed DSA against 6 HLA antigens: A1, B8, B35, DR13, DR15 and DQ6, with respective MFI of 10,000, 13,000, 4700, 10,000, 4000 and 21,000 (Fig. 3, left panel). Insulin could be stopped 10 days after the 3rd infusion. Primary graft function was optimal (i.e. ≥ 7). During the whole follow-up, no de novo DSA emerged. The recipient had no positive autoantibodies, from baseline to the end of follow-up. The patient remained insulin-independent for more than 7 years. At 5 years, her β-score started to decrease from 7 to 3, and at 10 years, she is still with a functioning graft (fasting C-peptide at 1.0 ng/ml).

Patient#2 was a 37-year-old woman, with a 36 year-history of type 1 diabetes (Fig. 3, right panel). She benefited from a first infusion of 3686 IEQ/kg with Protocol A immunosuppressive regimen. The 2nd injection was performed two months later of 3197 IEQ/kg. The 3rd islet infusion was finally performed one month later, with 4460 IEQ/kg. The patient had preformed DSA against the two first donors, with DSA targeting the DR15 antigen (1st donor), the DR13 antigen (2nd donor) and the DR9 antigen (2nd donor), with respective MFI values of 2500, 2800 and 2000. She did not develop any de novo DSA during the whole follow-up. Insulin could be stopped 1 month after the 3rd infusion. Primary graft function was optimal. At baseline, the recipient had no positive autoantibodies, and no autoantibodies emerged during follow-up. She remained insulin-independent for 93.5 months, then the β-score started to decrease with time, with respective values of 3, 1 and 0 at 96, 108 and 120 months. Graft loss was considered and the immunosuppressive regimen was withdrawn.

Impact of early de novo Donor Specific Antibodies with high or low fluorescence intensities.

**Figure 4:**
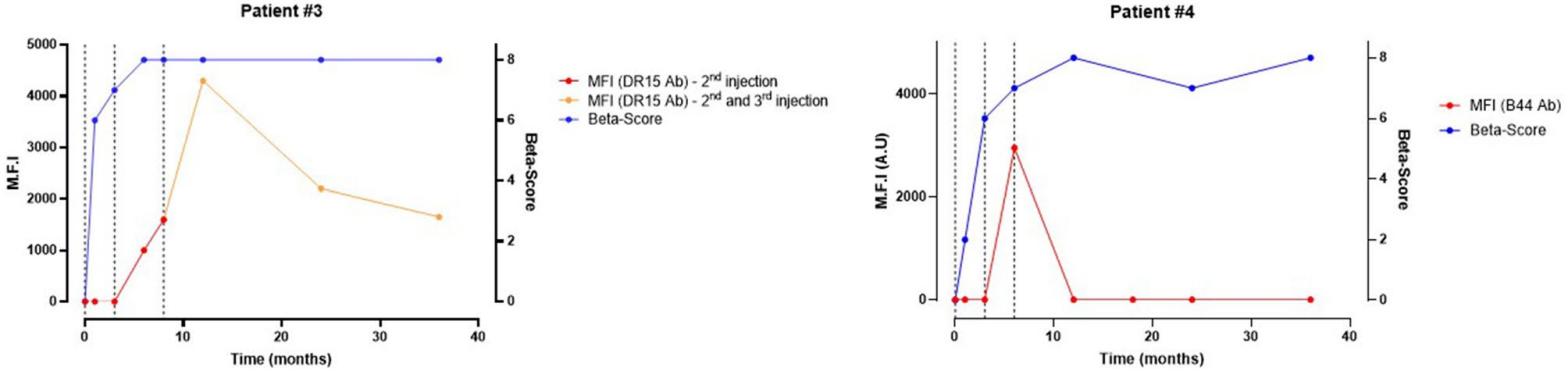
Evolution of β-Score over time in type 1 diabetic pancreatic islet recipients presenting with high or low early de novo Donor Specific Antibodies. Patient#3 (left panel) presented at 3 months post-injection with high-MFI de novo DSA targeting the 2nd islet infusion which became the preformed DSA of the 3rd infusion. Patient#4 (right panel) presented with a low-MFI early and transient DSA targeting the 3rd islet infusion, also at 3 months post-injection. Vertical dash lines represent every pancreatic islet injection. *Ab* Antibody; *MFI* Mean-Fluorescence intensity.

Patient#3 was a 56-year-old woman, with a 41 year -history of type 1 diabetes (Fig. 4, left panel). She benefited from a first infusion of 4277 IEQ/kg. Immunosuppressive regimen consisted in Protocol B. At the time of the first transplantation, she had two preformed DSA targeting the antigens A29 (MFI = 2000) and DR7 (MFI = 1500). The 2nd injection was performed 4 months later of 3324 IEQ/kg. At the time of the second transplantation, she had one preformed DSA targeting the A1 antigen (MFI = 7300). Between the 2nd and the 3rd infusion she developed de novo DSA against the 2nd injection (antigen DR15, MFI = 1500). The 3rd islet infusion was finally performed five months later, with 5269 IEQ/kg. She had then one preformed DSA against the 3rd donor, induced by the 2nd infusion, targeting the DR15 antigen. The MFI of the DR15 DSA continued to rise up to 4300, however no clinical symptoms were observed. Glycemia remained steady and insulin could be stopped. Primary graft function was optimal. Considering auto-antibodies, the patient was positive at baseline for anti-ICA antibodies. She became transiently weakly positive for anti-IAA and anti-GAD after transplantation and here ICA antibodies increased slightly after every islet infusion. Yet after 12 months post-transplantation, all the autoantibodies were negative until the end of follow-up. At the end of follow-up (36 months) she was still insulin-independent with a functional graft, a β-score of 8 and HbA1c level of 5.5%.

Patient#4 was a 61-year-old woman, with a 23 year-history of type 1 diabetes (Fig. 4, right panel). She benefited from a first infusion of 3080 IEQ/kg with Protocol A immunosuppressive regimen (daclizumab + tacrolimus + sirolimus). A 2nd injection of 4994 IEQ/kg was performed 3 months later. The 3rd islet infusion was finally performed four months later, with 4049 IEQ/kg. The patient had no preformed DSA against any of the donors at the time of each islet injection. HLA antibody screening was negative. Insulin could be stopped 1 month after the 2nd infusion. Primary graft function was optimal. One month after the 3rd infusion, a de novo DSA emerged targeting the B44 antigen from the 3rd infusion, with a MFI of 2950. No decrease in tacrolimus or sirolimus trough levels was found. One month later, the DSA disappeared, as the Luminex screening was negative again. Autoantibodies remained negative from baseline to the end of follow-up. At three years (end of follow-up), the patient remained insulin-independent, with a functional graft, a β-score of 7 and a HbA1c of 6.1%.

### Significance of late de novo donor specific antibodies

**Figure 5:**
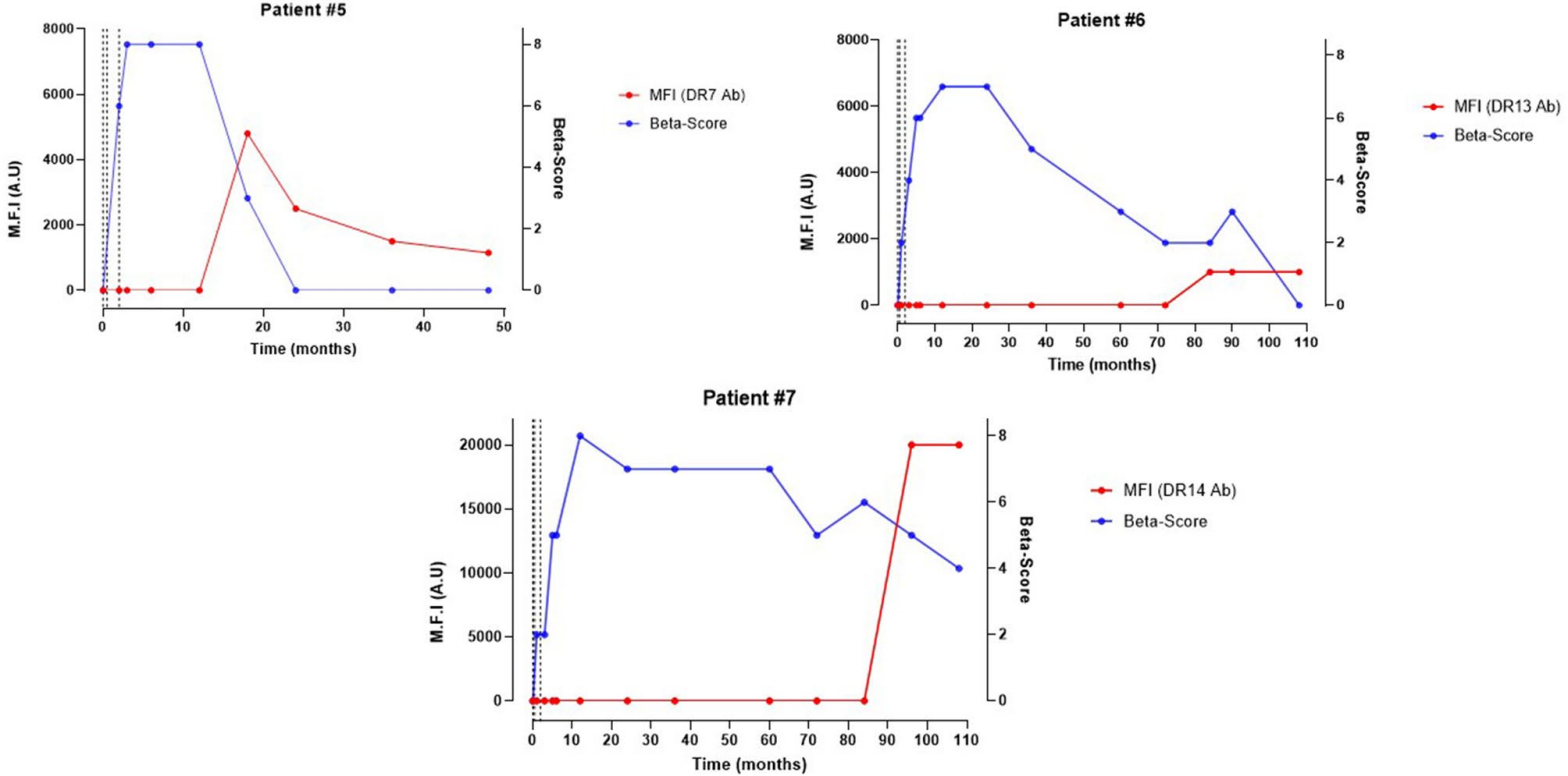
Evolution of β-Score over time in type 1 diabetic pancreatic islet recipients presenting with late de novo Donor Specific Antibodies. Patient#5 (top left panel), Patient#6 (top right panel) and Patient#7 (bottom panel) presented with late de novo DSA at 17 months, 77 and 99 months, respectively. Vertical dash lines represent every pancreatic islet injection. *Ab* Antibody; *MFI* Mean-Fluorescence intensity.

Patient#5 was a 58-year-old woman, with a 39-year history of type 1 diabetes. She benefited from two infusions, with a two-day interval, of 3010 IEQ/kg and 5498 IEQ/kg and of a 3rd islet infusion performed two months later, with 3288 IEQ/kg. The patient had no preformed DSA. Insulin could be stopped 11 days after the 3rd infusion. Primary graft function was optimal, with a β-score of 8. At 15 months, she was hospitalized for unsteady glycemia, with peaks between 2.0 and 3.0 g/L. She self-reported events of non-compliance to her immunosuppressive regimen in a context of mild cognitive dysfunction, even though no gaps in the tacrolimus trough levels could be found (Supplemental Fig. 3). HLA antibody screening revealed de novo DSA targeting 3 antigens of her first infusion, i.e. A3, A23 and DR7. MFI were 4000, 2500 and 4800 respectively. Her β-score dropped from 8 at 12 months to 3 at 18 months (Fig. 5, left top panel). According to the emergence of DSA, rejection was considered and insulin treatment was started again. Concomitantly, we observed the emergence of anti-GAD antibodies, negative from baseline to 12 months, positive at 18 months (20.9 U/ml) until the end of follow-up (Supplemental Fig. 4). The other autoantibodies remained undetectable. Severe hypoglycemic events occurred again at 21 months, mainly because of cognitive dysfunction and difficulties to manage the insulin treatment. Graft loss was considered at 3 years and all immunosuppressive therapeutics were stopped.

Patient#6 was a 57-year-old woman, with a 54 year-history of type 1 diabetes (Fig. 5, right top panel). She benefited from a first infusion of 4575 IEQ/kg with Protocol A immunosuppressive regimen. The 2nd injection was performed 3 months later of 4377 IEQ/kg. The 3rd islet infusion was finally performed two months later, with 4870 IEQ/kg. The patient had no preformed DSA against any of the donors at the time of each islet injection. HLA antibody screening was negative. Insulin could be stopped 1 month after the 3rd infusion. Primary graft function was optimal with a β-score of 7 one month after the last infusion. At 18 months, sirolimus was switched to mycophenolate mofetil because of proteinuria and focal and segmental glomerulosclerosis associated with mTOR inhibitors. Her β-score remained stable up to 3 years with a β-score of 7. However, the β-score started to decline at 3 years, and she had to start insulin again. The β-score continued to decrease to 2 at 6 years, with a persisting graft function (fasting C-peptide of 1.0 ng/ml). At 6 years, a DSA targeting the DR11 antigen from the 3rd infusion emerged with a MFI of 1000. Considering auto-antibodies, none of them were detectable at baseline. At 8 years, we observed the appearance of anti-GAD antibodies (13 U/ml), negative from baseline until then, which remained positive until the end of follow-up (Supplemental Fig. 4). Graft function continued then to decrease slowly with a stimulated C-peptide of 0.59 ng/ml at 7 years (β-score of 2), 0.19 ng/ml at 8 years (β-score of 2) and 0.24 ng/ml at 9 years (β-score of 0).

Patient#7 was a 56-year-old man, with a 23-year history of type 1 diabetes (Fig. 5, bottom panel). He benefited from a first infusion of 5031 IEQ/kg with Protocol B immunosuppressive regimen. The 2nd injection of 5843 IEQ/kg was performed 5 months later. The 3rd islet infusion was finally performed one month later, with 3413 IEQ/kg. The patient had no preformed DSA against any of the donors at the time of each islet injection. Insulin could be stopped 1 month after the 3rd infusion. Primary graft function was optimal with a β-score of 7 one month after the last islet infusion. His β-score remained steady around 7 until year 5, when it started slowly to fluctuate between 5 and 6, yet the patient was still insulin-independent. At 99 months, a gap in the tacrolimus trough levels was observed, concomitant to a change of Tacrolimus dosage (Supplemental Fig. 3, right bottom panel). A few weeks later, two DSA targeting the DR14 antigen from the 2nd infusion and the DQ5 antigen from the 3rd infusion emerged, with respective MFI of 20,000 and 19,000. At baseline, the recipient had no positive autoantibodies, and no autoantibodies emerged during follow-up. At the end of follow-up (108 months), he remained still insulin-independent, yet with a patent decrease of his β-score from 6 at 7 years (fasting C-peptide of 1.26 ng/ml, glycemia of 4.62 mmol/L, HbA1c of 6.3%, insulin-free) to 4 at 9 years (fasting C-peptide of 0.8 ng/ml, glycemia of 8.965 mmol/L, HbA1c of 7.1%, insulin-free).

### HLA antibodies in the context of Islet-after-kidney transplantation

In our cohort, there were 7 islet-after-kidney recipients with a median time of follow-up of 60 months (48–78). The median time between kidney transplantation and islet transplantation was 28.2 months (21.2–44.2). Five recipients received thymoglobulin as immunosuppressive inductive agent during kidney transplantation, whereas the two others received basiliximab. For islet transplantation, all of them benefited from an immunosuppressive regimen according to the Edmonton protocol, with a conversion from mycophenolate mofetil to sirolimus before islet transplantation. We compared tacrolimus and sirolimus trough levels between ITA and IAK. Overall, we did not find any significant difference between IAK and ITA considering tacrolimus trough levels (fixed effect between ITA and IAK recipient: −0.74, 95% CI: from −1.79 to 0.30, *p* = 0.16), or sirolimus trough levels (fixed effect between ITA and IAK recipient: −0.87, 95% CI: from −2.84 to 1.1, *p* = 0.36) (Supplemental Fig. 2B).

None of the islet-after-kidney recipients presented preformed or de novo DSA against the pancreatic islets or the kidney graft. None of them lost their kidney graft during follow-up.

## Discussion

We report here an illustrative case-series with seven clinical cases dealing with the impact of DSA on islet transplantation outcomes. Patient#1 and Patient#2 were transplanted with preformed DSA and presented favorable outcomes whatever the DSA’s MFI were. Patient#3 presented with both preformed DSA and early high-MFI de novo DSA and Patient#4 with early low-MFI de novo DSA. Both presented with favorable metabolic outcomes at 3 years post-transplantation. Finally, only Patients #5, #6 and #7 presented with unfavorable metabolic outcomes, concomitantly to the occurrence of late de novo DSA. Only islet-transplantation-alone recipients had de novo DSA in our cohort, as islet-after-kidney recipients remained DSA-free during their whole follow-up.

In the general context of solid organ transplantation, DSA are the leading cause of graft failure. Preformed DSA constitutes a relative contraindication to transplantation^17^, especially for kidney transplantation^8^. De novo DSA is a key component of antibody-mediated rejection, resulting in microvascular inflammation^7^, and is associated with poor outcomes. Considering islet transplantation, the prevalence of preformed DSA has been reported between 0 and 50%, whereas the prevalence of de novo DSA has been reported between 10 and 50%, and seems to be associated with immunosuppression withdrawal^12,18–21^. Considering preformed DSA, Piemonti et al. presented one of the largest cohorts of islet transplantation with a complete screening of HLA antibodies during follow-up. Twenty-nine recipients out of 59 had preformed DSA and 24 patients de novo DSA. Preformed DSA were not associated with an increased risk of graft failure. On the contrary, de novo DSA were associated with reduced graft survival compared with no DSA. The management of preformed DSA, and the question whether it should contraindicate or not any islet transplantation, is thus critical specifically in the case of islet transplantation, where three donors are generally required. Indeed, it is noteworthy that Patient#1 in our case-series could remain insulin-independent for several years, despite the presence of DSA against the three donors, with several DSA having MFI values over 10,000. In other organ-transplant settings, the transplantation may have probably been proscribed, or desensitization protocols would have been discussed to lower the rate of anti-HLA antibodies and facilitate the access to transplantation.

Considering de novo DSA, the task to decipher their impact on islet transplantation outcomes is more complex. Brooks et al. reported 16 patients transplanted with 26 islet infusions^20^. Five of them developed de novo DSAs, associated with graft failure at 12 months. This suggested, as Piemonti et al. reported^12^, a potential harmful effect of de novo DSA. Later on however, Pouliquen et al. reported 42 recipients^13^, including one patient with preformed DSA, and 12 who developed de novo DSA. The median time of de novo DSA appearance was 25.5 (9.6–85.8) months. They did not find any association between DSA and the risk of graft loss, even with MFI over 6000. In our cohort, the time of DSA occurrence, more than the MFI, seem to have an impact, as only late DSA were associated with the decrease of β-score. To help answer the question of the impact of de novo DSA on islet graft function, the answer may come from basic science. Chen et al. recently provided evidence that allogeneic islets may be resistant to DSA-mediated rejection despite the fact that DSA can destroy islet cells in vitro, using murine experimental models^22^. The vascular sequestration of DSAs in a donor-recipient chimeric neo-endothelium may protect islets from DSAs. These results may suggest that rejection in the context of islet transplantation may be a cellular-mediated rejection, as suggested in previous studies^10^. One can hypothesize then that preformed and early de novo DSA may not be harmful as they would be trapped into the recipient’s vasculature. On the contrary, late de novo DSA may be a biological marker of rupture of tolerance and cellular rejection. This is highlighted by the fact that two recipients out of three with late de novo DSA had a context of immunosuppressive regimen mismanagement (Patient#5 and Patient#7). Rupture of tolerance might also be suggested, as two recipients showed signs of autoimmunity emergence with anti-GAD antibodies appearing concomitantly or few shortly after alloantibodies. This drives the general question of the determinants of allograft dysfunction in islet transplantation. In the first days of islet transplantation, an important and rapid tissue loss is associated with the exposure of islets to human blood which triggers an “instant blood mediated inflammatory reaction” (IBMIR), characterized by the activation of both coagulation and complement pathways^2^. After a few months post-transplantation, the metabolic response improves to the highest at approximately 12 months post-transplant, as depicted by Ryan et al. followed then by a decrease in metabolic function over time^16^. This metabolic function decrease is most likely multifactorial, including acute and chronic alloimmune rejection, autoimmunity, chronic fibrosis due to a non-physiological environment, and drug-induced toxicity of immunosuppressive drugs^23,24^. Indeed, both tacrolimus and sirolimus have diabetogenic properties. Yet, conclusions are often drawn from basic science and murine models and there is a clear lack of human post-transplantation histopathological studies. Indeed, considering the risk associated with a liver biopsy and its low yield^25^, the main histopathological studies come from autopsy or transplantectomy cases. Immune cell infiltration into islets has been reported^25^ as well as amyloid deposits^26^, without any correlation possible between these injuries and a clinical phenotype. All the reported samples were obtained randomly, and for now, no biopsy samples have been reported from an islet transplant recipient suspected of rejection. Considering the absence of histopathology-based rejection classification in the islet transplantation field, further clinical-based investigations are required to assess these statements, in particular tools to measure cellular reactivity against islets^9,27^.

To end with, the last challenge consists in the complexity of multiple allogenic stimulation in islet-after-kidney transplantation. First, the Edmonton protocol requires often an immunosuppressive regimen switch in order to include Sirolimus, which might be associated with an increased risk de novo DSA and rejection. However, none of our patients experienced immunological complications from the immunosuppressive switch. Second, the immune sensitization induced by several allogenic stimuli may cross-react with the underlying kidney graft. In case of similar HLA typing between the kidney graft and the islet infusions, one de novo DSA may recognize both type of donors and induce rejection. However for now, all clinical data have proven islet-after-kidney to be safe, with no harm of several allogenic stimuli over the underlying kidney, once the switch is done progressively in carefully selected patients^28,29^.

Our study carries several limits, as we analyzed retrospective data which brings the risk of information loss in a small cohort. We were limited to a single-center analysis, as the management of immunosuppressive therapies differs from one center to another, involving notably the discontinuation of the immunosuppressive regimen in islet-transplantation-alone recipients in some centers^13^. Furthermore, islet transplantation remains a rather rare transplantation, so we lack of a sufficient volume to draw solid conclusions. Yet, regulatory authorities in many countries are moving towards a global increase of transplanted recipients, as islet transplantation is reimbursed in some countries, and is authorized for reimbursement in France since 2021 and its status is slowly improving in the U.S^30^. Considering the suspected impact of DSA and the prevalence of de novo DSA in islet transplantation, large-scale registries are needed to decipher the complexity and interrelationship between DSA, autoimmunity and allograft loss. Furthermore, as de novo DSA is a time-dependent event, and thus its impact may be influenced by the length of follow-up between transplantation and the event itself, dedicated statistical models should be built for such a purpose. Currently, no large-scale registry exists in islet transplantation dealing with the specific issue of donor specific antibodies, and further research is warranted. This study is then meant to reflect the experience of our group regarding the difficulties related to allogenicity and immunosuppressive management from a physician’s perspective to help decision making.

## Conclusion

Clinical-based trajectories in a single-center experience reveal that islet transplantation with preformed DSA, even with high MFI values, may be associated with favorable outcomes. On the contrary, de novo DSA, and especially late de novo DSA, may be associated with allograft loss.

## Supplementary Information

Supplementary Information.

## Data Availability

The data that support the findings of this study are available from the corresponding author upon reasonable request.

## Abbreviations

DSA: Donor specific antibodies
IAA: Insulin auto antibodies
IAK: Islet-after-kidney
ICA: Islet cell autoantibodies
IEQ/kg: Islet-equivalent/kg
ITA: Islet-transplantation alone
GAD: Glutamic acid decarboxylase
MFI: Mean fluorescence intensity
