# Supplementary material for: The challenge of HLA donor specific antibodies in the management of pancreatic islet transplantation: an illustrative case-series
Source: Sci Rep. 2022 Jul 21;12:12463. doi: 10.1038/s41598-022-16782-3 (PMC9304358; doi:10.1038/s41598-022-16782-3)
Supplement: Supplementary file 1 — Supplementary Information. [file 41598_2022_16782_MOESM1_ESM.pdf]

The challenge of HLA donor specific antibodies in the management of pancreatic islet transplantation: an illustrative case-series.

Mehdi Maanaoui[1,2$*], MD, Mikael Chetboun[2,3$], MD, Isabelle Top[4], MD, Vincent Elsermans[4], MD, Julie Kerr-Conte[2,5], PhD, Kristell Le Mapihan[2,6], MD, Frederique Defrance[2,6], MD, Valéry Gmyr[2,5], PhD, Thomas Hubert[2], PhD, Myriam Labalette[4], MD, PhD, Marc Hazzan[1§], MD, PhD, Marie-Christine Vantyghem[2,6§], MD, PhD, François Pattou[2,3§], MD, PhD.

[1] CHU Lille, Department of Nephrology, F-59000, Lille, France

[2] Univ. Lille, Inserm, CHU Lille, Institut Pasteur Lille, U1190 - EGID, 59000, Lille, France.

[3] CHU Lille, Department of General and Endocrine Surgery, F-59000, Lille, France

[4] CHU Lille, Service d'Immunologie, F-59000, Lille, France

[5] Plateforme de Biothérapie, CHU Lille, 59000, Lille, France.

[6] CHU Lille, Department of Endocrinology, Diabetology, and Metabolism, F-59000, Lille, France

[$] shares co-first authorship

[§] shares co-last authorship

Running title: Islet transplantation and Donor Specific Antibodies.

Corresponding author:

Mehdi MAANAOUI, Service de Néphrologie, Hôpital Huriez, CHU de Lille,
59037 Lille, France. Tel: +33 320444034

E-mail: mehdi.maanaoui@gmail.com

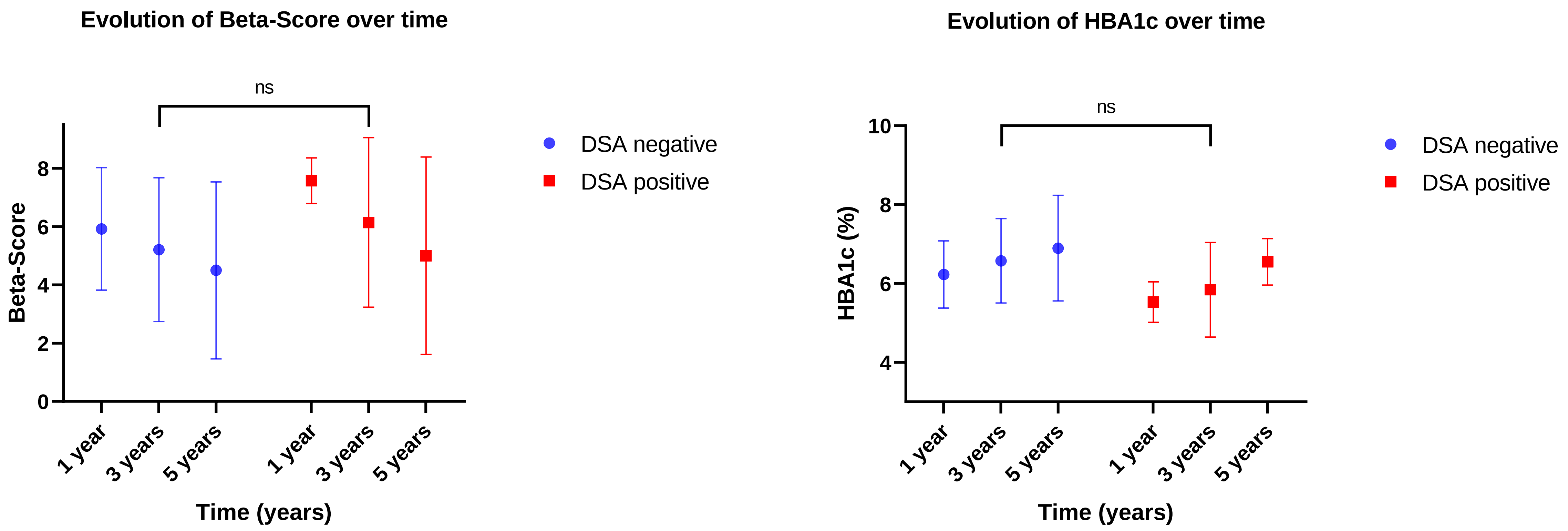

Supplemental Figure 1. Evolution over time of Beta-Score and HBA1c trough levels in islet transplant recipients. There was no significant effect of the presence of DSA on the evolution of Beta-Score at 1 year, 3 years and 5 years (fixed effect between DSA negative and positive recipient: -1.07, 95% CI: from -3.05 to 0.92, p = 0.16), or the evolution of HBA1c (fixed effect between DSA negative and positive recipient: 0.55, 95% CI: from -0.26 to 1.36, p = 0.18). DSA = Donor Specific Antibody, ns = non significant. Results are shown with mean and standard deviation.

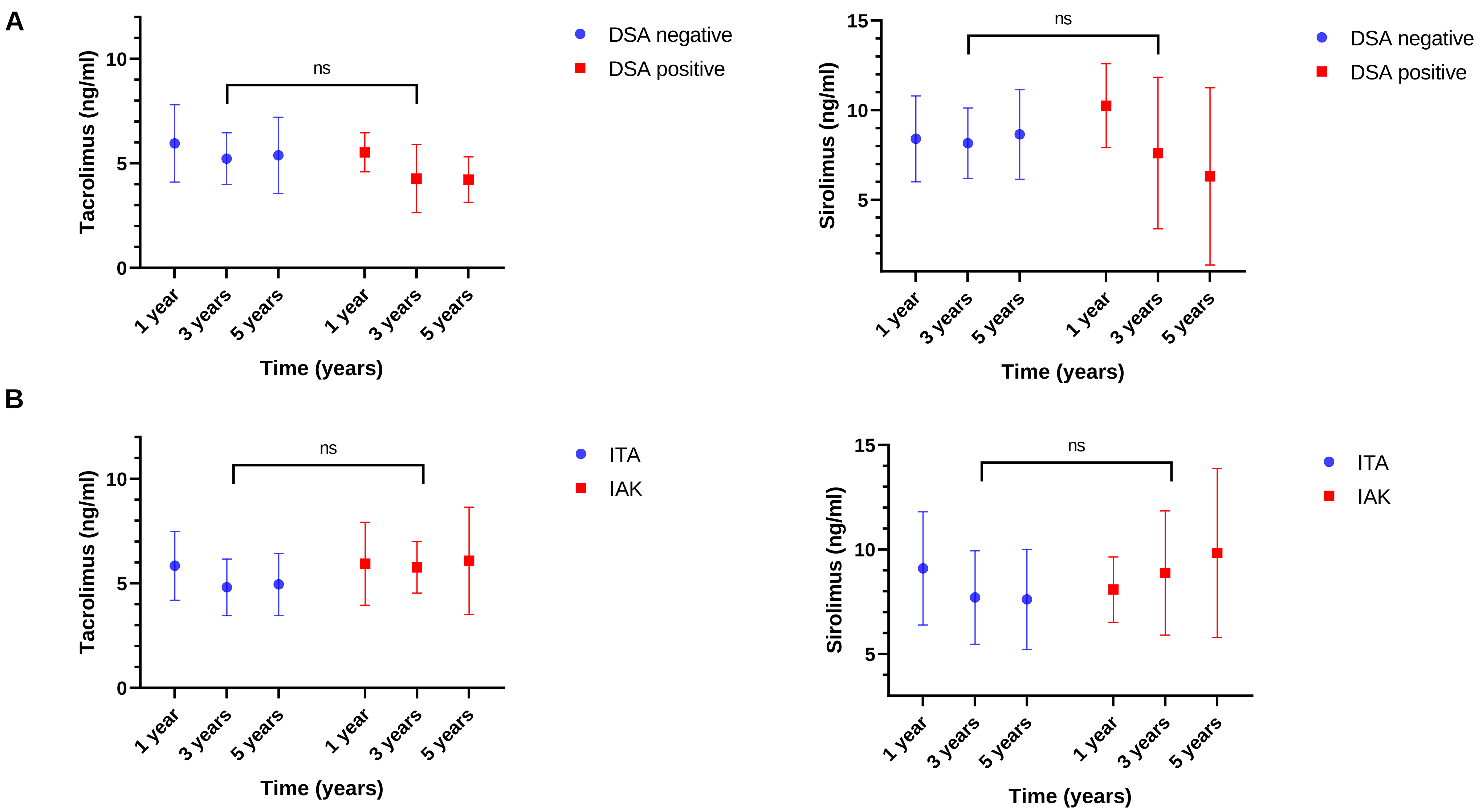

Supplemental Figure 2. Evolution over time of tacrolimus and sirolimus trough levels in islet transplant recipients. (A) There was no significant difference between DSA negative and positive considering tacrolimus trough levels (fixed effect between DSA negative and positive recipient: 0.80, 95% CI: from -0.23 to 1.84, p = 0.12), or sirolimus trough levels (fixed effect between ITA and IAK recipient: 0.40, 95% CI: from -1.76 to 2.56, p = 0.70). **(B)** There was no significant difference between IAK and ITA considering tacrolimus trough levels (fixed effect between ITA and IAK recipient: -0.74, 95% CI: from -1.79 to 0.30, p = 0.16), or sirolimus trough levels (fixed effect between ITA and IAK recipient: -0.87, 95% CI: from -2.84 to 1.1, p = 0.36). DSA = Donor Specific Antibody, IAK = Islet-After-Kidney, ITA = Islet-Transplantation Alone, ns = non significant. Results are shown with mean and standard deviation.

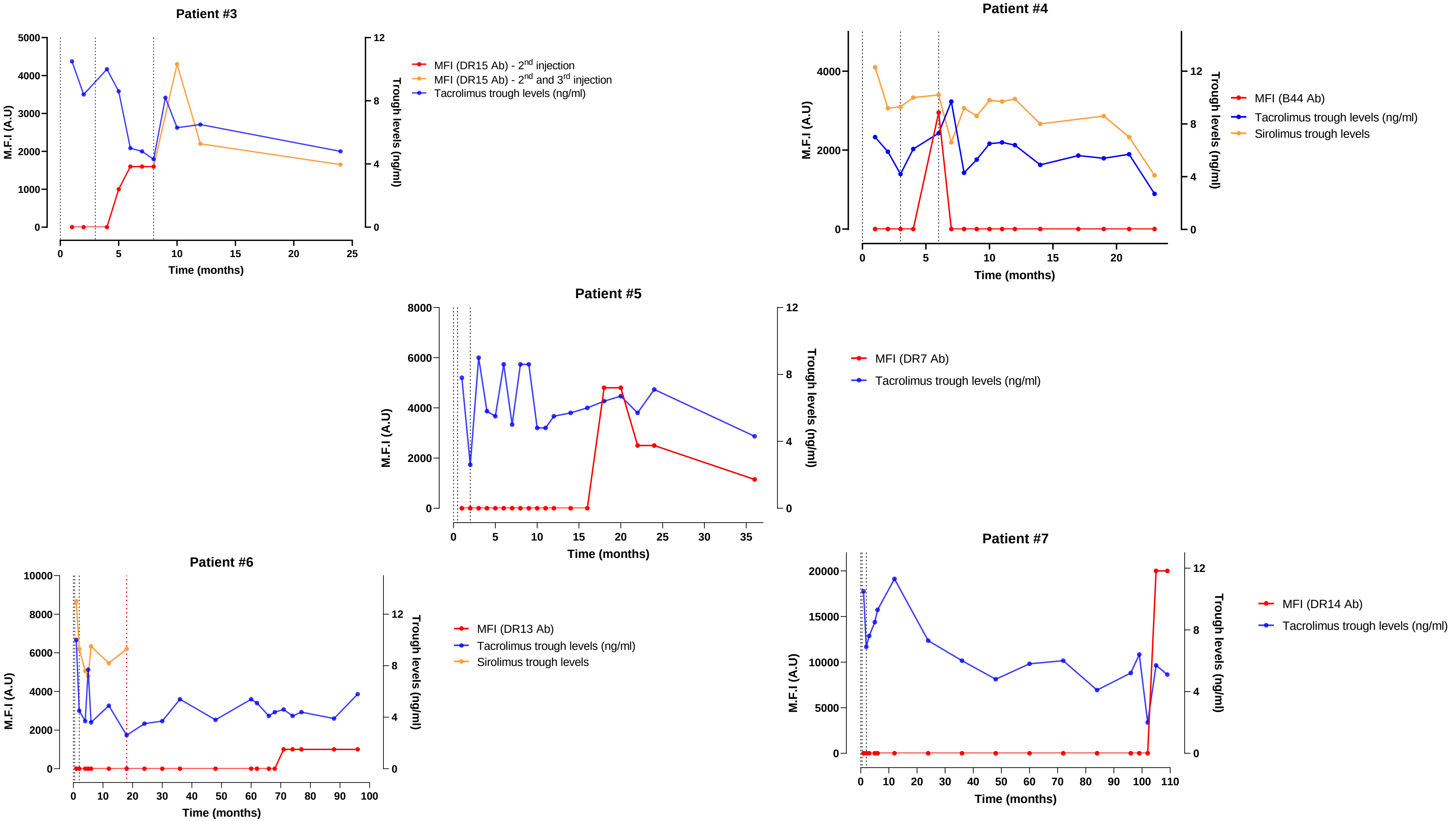

Supplemental Figure 3. Evolution over time of Tacrolimus trough levels in recipients presenting with de novo DSA. Vertical dash lines represent every pancreatic islet injection. Ab: Antibody; MFI: Mean-Fluorescence intensity.

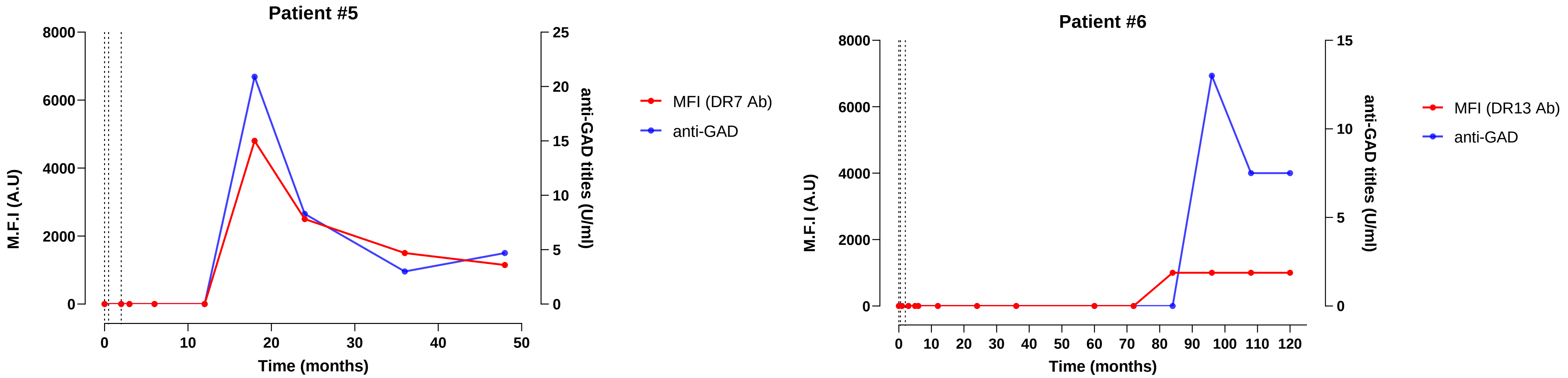

Supplemental Figure 4. Evolution over time of autoimmune antibodies in two patients with concomitant allo- and autoimmune resurgence. Vertical dash lines represent every pancreatic islet injection. Ab: Antibody; GAD:Glutamic Acid Decarboxylase; MFI: Mean-Fluorescence intensity.

Supplemental Table 1. Detailed presentation of HLA characteristics and antibodies in the recipients presenting specific clinical-based trajectories.

	Patient#1	Patient#2	Patient#3	Patient#4	Patient#5	Patient#6	Patient#7
Type of recipient	ITA	ITA	ITA	ITA	ITA	ITA	ITA
Recipient HLA typing	A24/A30-B13/B18-DR3/DR4-DQ2/DQ8	A2/A24-B65/B18-DR3-DQ2	A2-B18-DR3-DQ2	A2/A29-B65/B18-DR1/DR11-DQ5/DQ7	A2/A32-B8/B18-DR3-DQ2	A2/A3-B7/B60-DR4-DQ8	A3/A26-B35-DR1/DR4-DQ5/DQ8
1st infusion							
HLA typing	A2-B62/B44-DR4-DQB3	A2/A68-B7/B72-DR15/DR3-DQ6/DQ2	A3/A23-B44/B51-DR11/DR7-DQ7/DQ2	A1/A23-B7/B8-DR17-DQ2	A29/A32-B13/B35-DR7/DR13-DQ2/DQ6	A3/A68-B44/B49-DR4/DR7-DQ2/DQ3	A2/A29-B44/B51-DR4/DR7-DQ2/DQ7
preformed DSA: antigen, MFI	A2, 11000<br>B62, 1330<br>B44, 1700<br>DR4, 8350	DR15, 2500	0	0	0	0	A29, 2000<br>DR7, 1500<br>B44, 600
de novo DSA until end of follow-up: antigen, maximum MFI (time of emergence in months)	0	0	A3, 4000 (15 mo.)<br>A23,2500 (15 mo.)<br>DR7, 5000 (15 mo.)	0	DR13, 1000 (90 mo.)		0
2nd infusion							
HLA typing	A1/A24-B8/B40-DR2/DR4	A23-B41/B44-DR13/DR9-DQ6/D3	A2/A29-B61/B44-DR4/DR8-DQ7/DQ4	A2/A29-B7/B51-DR13/DR16-DQ6/DQ5	A3/A29-B7/B44-DR16/DR3-DQ5/DQ2	A3/A11-B7/B18-DR14-DQ5	A1/A31-B51/B63-DR13/DR15-DQ6
preformed DSA: antigen, MFI	A1, 10000<br>B8, 13000<br>B62, 2000<br>DR4, 7500	DR13, 2800<br>DR9, 2000	0	0	0	0	A1, 9500
de novo DSA until end of follow-up: antigen, maximum MFI (time of emergence in months)	0	0	0	0	0	DR14, 20000 (100 mo.)	DR15, 4300 (3 mo.)
3rd infusion							
HLA typing	A1-B8/B35-DR15/DR13-DQB6/DQ6	A1/A2-B8/B57-DR3/DR7-DQ2/DQ3	A2/A26-B27/B62-DR15/DR4-DQ6/DQ8	A2/A24-B7/B44-DR4/DR10-DQ7/DQ5	A3/A24-B18/B4-DR11/DR17-DQ7/DQ2	A2/A11-B44/B55-DR4/DR16-DQ5/DQ7	A2/A31-B7/B44-DR15/DR4-DQ6/DQ8
preformed DSA: antigen, MFI	A1, 10000<br>B8, 13000<br>B35, 4700<br>DR13, 10000<br>DR15, 4000<br>DQ6, 21000	0	0	0	0	0	B44, 1000<br>DR15, 1600
de novo DSA until end of follow-up: antigen, maximum MFI (time of emergence in months)	0	0	0	B44, 2950 (3 mo.)	DR11, 1000 (76 mo.)	DQ5, 19000 (99 mo.)	0

DSA= Donor Specific Antibody; ITA = Islet Transplantation Alone; IAK = Islet-After-Kidney transplantation; HLA = Human Leukocyte Antigen; MFI = Mean Fluorescence Intensity; mo.= months; N/A= non appliable;
